# Synthesis and photocatalytic activity of CeO_2_-NbC-x catalysts for degrading oxytetracycline in aquaculture water bodies

**DOI:** 10.3389/fchem.2026.1778672

**Published:** 2026-03-04

**Authors:** Fanggang Hong, Langtao Gao

**Affiliations:** College of Resources and Environment, Jilin Agricultural University, Changchun, China

**Keywords:** CeO2@NbC, molten salt method, oxytetracycline hydrochloride, photocatalytic mechanism, polyacrylamide gel method

## Abstract

The aquaculture water body is rich in antibiotics, which has caused great harm to the natural environment. It is urgent to adopt special means to degrade antibiotics from the source and reduce their impact on human production and life. In this study, the CeO_2_@NbC (CeO_2_-NbC-x (where x denotes the wt% of NbC)) catalyst was synthesized by the molten salt method, polyacrylamide gel method and heat treatment technology. Multiple characterization methods confirmed that there were no other impurities in the CeO_2_-NbC-x catalyst, and a special contact was formed between the CeO_2_ and NbC interfaces. The CeO_2_-NbC-x catalyst exhibits high ultraviolet and visible light optical absorption coefficients. Using oxytetracycline hydrochloride as the target pollutant, the effects of catalyst concentration, pollutant concentration and pH value on the photocatalytic activity of the CeO_2_-NbC-x catalyst were investigated. When the catalyst concentration, pollutant concentration and pH value were 2 g/L, 75 mg/L, and 5, respectively, the degradation percentage of CeO_2_-NbC-x catalyst reached 99%. Based on capture experiments and band arrangement theory, a new photocatalytic mechanism of the CeO_2_-NbC-x catalyst has been proposed. This research provides new ideas for the application of wide bandgap semiconductors couple semiconductors with metallic properties in the field of photocatalysis.

## 1 Introduction

With the rapid development of the global economy and the increasing demand for aquatic products, a large amount of antibiotics is needed to ensure the survival rate and yield of aquatic products (Lulijwa et al., 2020). However, only a small amount of antibiotics used in aquaculture bases can be absorbed by aquatic products, while the majority are discharged into the water environment along with feces (Limbu et al., 2021). A large accumulation will cause great harm to the water environment, thereby affecting people’s lives and health. Therefore, degrading antibiotics from the source helps reduce environmental pollution and thereby improve people’s quality of life.

At present, the main methods for degrading antibiotics or other pollutants include adsorption, electrocatalysis, photocatalysis, piezoelectric catalysis and biodegradation, etc (Ali et al., 2025; Choudhary et al., 2021; Dutta et al., 2021; Ma et al., 2025; Mangla et al., 2022; Shi et al., 2024; Srivastava, 2026; Sun et al., 2024; Zhang et al., 2024). Among these methods, photocatalytic technology, which degrades pollutants by means of sunlight, is hailed as an efficient and green technology (Baaloudj et al., 2021; Du et al., 2026; Soorya et al., 2025; Yang et al., 2025; Zulfiqar et al., 2024). Designing appropriate photocatalysts for the degradation of antibiotics is currently a research hotspot (Mousa et al., 2025). Cerium dioxide (CeO_2_) is an outstanding photocatalyst with extensive applications in the field of photocatalysis due to its high charge carrier migration rate, high vacancy concentration, and excellent thermal and chemical stability (Ansari et al., 2025; Cheng et al., 2025; Deng et al., 2025). However, the wide bandgap value of the CeO_2_ enables it to respond only to ultraviolet light, which accounts for only 3% of sunlight, greatly limiting its application in the field of photocatalysis (Nadjia and Abdelkader, 2025). Therefore, focusing efforts on improving the visible light response capability of the CeO_2_ can help broaden its application in the field of photocatalysis. Researchers have made great efforts in the construction of the CeO_2_-based heterostructures to enhance their visible light photocatalytic activity (Li et al., 2025; Luo et al., 2026; Zhang et al., 2025). From this perspective, constructing a special heterojunction is conducive to enhancing the visible light photocatalytic activity of the CeO_2_.

Niobium carbide (NbC) is a semiconductor material with metallic properties and has great application prospects in the field of photocatalysis due to its high charge transfer and separation capabilities, high stability, and high charge carrier transport capacity (Chen et al., 2013). Most researchers have coupled NbC with other semiconductors or C, etc., which can effectively photocatalytic degrade different pollutants (Gupta et al., 2019; Gupta and Pandey, 2018; Zhang et al., 2022). Inspired by this, coupling NbC with CeO_2_ to form a CeO_2_@NbC (CeO_2_-NbC-x (where x denotes the wt% of NbC)) catalyst is expected to exhibit high visible light photocatalytic activity, but no reports have been made yet. Therefore, it is of great significance to synthesize CeO_2_-NbC-x catalysts by special methods and study their photocatalytic activities.

In this paper, we propose the use of the molten salt method, polyacrylamide gel method and heat treatment technology to synthesize CeO_2_-NbC-x catalyst. A special heterojunction was confirmed to have formed between CeO_2_ and NbC through various characterization methods. The visible light response capability of the CeO_2_ was significantly improved after NbC was coupled with CeO_2_. Taking oxytetracycline hydrochloride (OTC-HCl) as the research object, the effects of different NbC contents, catalyst concentrations, pollutant concentrations and pH values on the photocatalytic activity of the CeO_2_-NbC-x catalyst were explored. Based on the experimental results, a reasonable photocatalytic mechanism of the CeO_2_-NbC-x catalyst was proposed.

## 2 Materials and methods

### 2.1 Preparation of NbC

**FIGURE 1:**
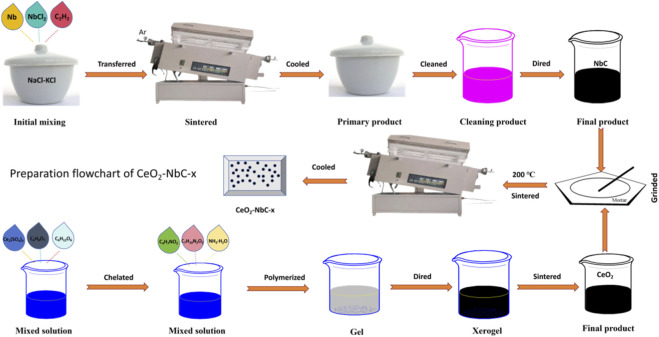
Preparation flowchart of the CeO_2_-NbC-x.

According to the chemical formula of the NbC, the precursors of Nb and C were weighed in equal molar ratios. Nb and NbCl_5_ were used as the Nb sources, and acetylene black (C_2_H_2_) powder was used as the C source. The eutectic molten salt of the NaCl-KCl was used as the reaction medium, with a mass ratio of 7 : 1 to the aforementioned precursor. Thoroughly mix Nb, NbCl_5_, C_2_H_2_ and NaCl-KCl and place them in a covered alumina crucible. Place the crucible in a tube furnace filled with argon gas and react at 900 °C for 2 h. After cooling to room temperature, take it out to obtain the primary product. Wash the primary product several times with deionized water to obtain the washed product. The cleaned product is dried in a drying oven at 120 °C for 24 h to obtain the final product (NbC). The corresponding preparation flowchart is shown in Figure 1. According to the experimental description, the following reactions (Equations 1, 2) can occur:

$$\text{Nb}+4\text{Nb}\left(V\right)\;\to \;5\text{Nb}\left(\text{IV}\right)\;\left(\text{in NaCl}-\text{KCl}\right)$$ (1)

$$5\text{Nb}\left(\text{IV}\right)+C\;\to \;4\text{Nb}\left(V\right)+\text{NbC}$$ (2)

In reaction Equation 2, since Nb(V) is generated on the right side, it will continue to react with the remaining Nb until it is exhausted. Therefore, the entire reaction process is equivalent to reaction Equation 3.

Nb+C → NbC (3)

The main purpose of adding molten salt is that the reaction system can react with Nb to form NbCl_5_ even in the absence of NbCl_5_ (Yan et al., 2019). The specific reaction (Equation 4) are as follows:

$$\text{Nb}+5{\text{Cl}}^{-}\;\to \;{\text{NbCl}}_{5}$$ (4)

### 2.2 Preparation of CeO_2_

The preparation of the CeO_2_ was performed by the most classic polyacrylamide gel method (Hu et al., 2018; Wang et al., 2021). There are two differences from the methods reported in the literature: First, the polymerization of acrylamide (C_4_H_7_NO_2_) and methylene diacrylamide (C_7_H_10_N_2_O_2_) needs to be initiated through temperature polymerization. Second, in some methods reported in the literature, the use of methylene diacrylamide is not required. Cerium sulfate (Ce_2_(SO_4_)_3_) was used as the Ce source, citric acid (C_6_H_8_O_7_) as the chelating agent, and glucose (C_6_H_12_O_6_) as the anti-gel collapse agent. After weighing them in the corresponding proportions, 6.0645 g Ce_2_(SO_4_)_3_, and 4.7282 g C_6_H_8_O_7_ were successively added to 50 mL of deionized water. Subsequently, 9.5958 g acrylamide and 1.9192 g methylene diacrylamide were added almost simultaneously. After the above reagents are completely dissolved, add ammonia water (NH_3_·H_2_O) to adjust the pH value of the solution to 5. After adjusting the pH value, heat up to 100 °C until a jelly-like gel is obtained. The obtained gel was placed in a drying oven and dried at 120 °C for 48 h to obtain black xerogel. The black xerogel was ground into fine powder and sintered at 900 °C in a tube furnace to obtain CeO_2_ nanoparticles. The corresponding preparation process is shown in Figure 1.

### 2.3 Preparation of CeO_2_-NbC-x

The NbC prepared in Section 2.1 and the CeO_2_ prepared in Section 2.2 were weighed into the corresponding powders in mass ratios of 25 wt% NbC, 50 wt% NbC and 75 wt% NbC, respectively, and then ground in a mortar for 2 h. The obtained samples were marked in sequence as CeO_2_-NbC-25, CeO_2_-NbC-50, CeO_2_-NbC-75. The mixture ground in a mortar is sintered at 200 °C for 5 h to obtain CeO_2_-NbC-x. The preparation flowchart of CeO_2_-NbC-x as shown in Figure 1.

### 2.4 Materials characterization

The phase structure of the CeO_2_, CeO_2_-NbC-25, CeO_2_-NbC-50, CeO_2_-NbC-75 and NbC was measured by a D8 ADVANCE X-ray powder diffractometer (XRD) with Cu Kα radiation. The surface morphology of the CeO_2_, CeO_2_-NbC-25, CeO_2_-NbC-50, CeO_2_-NbC-75 and NbC were observed by a field emission scanning electron microscopy (SEM) and transmission electron microscopy (TEM). The optical properties of the CeO_2_, CeO_2_-NbC-25, CeO_2_-NbC-50, CeO_2_-NbC-75 and NbC were analyzed by a UV-2450 type UV-visible spectrophotometer.

### 2.5 Photocatalytic experiments

To explore the photocatalytic activity of the CeO_2_-NbC-x catalyst, OTC-HCl was used as the target pollutant to complete the corresponding photocatalytic experiment. Before the photocatalytic experiment was performed, to eliminate the influence of adsorption on photocatalysis, a half-hour adsorption experiment was conducted. During photocatalysis, a 300 W xenon lamp is used as the light source to simulate sunlight. The experiment was carried out in a self-made reactor. The dosage of the catalyst is 0.5–3 g/L, the pollutant concentration is 25–100 mg/L, and the pH value is 1–7. These parameters are adjusted according to the experimental requirements. The specific details are mainly described in the discussion section of the photocatalytic results. After the photocatalytic experiment began, samples were taken every 10 min until the experiment was completed 60 min later. Each time, 5 mL was sampled. After sampling, the samples were first centrifuged and then the concentrations of contaminants before and after the reaction were measured using a 721 spectrophotometer, which were respectively recorded as C_0_ and C_t_. The corresponding degradation percentage (D%) can be described by Equation 5.

$$D\%=\left(\left(C_{0}-C_{t}\right)/C_{0}\right)\times 100$$ (5)

Where, C_0_ and C_t_ is the initial concentration and the concentration at time t of the OTC-HCl, respectively. To explore the contribution of free radicals in photocatalytic experiments, capture experiments were performed. The EDTA-2Na, IPA and BQ were used to capture the hole, hydroxyl radical and superoxide radical, respectively. The experimental process is similar to the photocatalytic experiment, except that 2 mol/L of capture agent is added to the reaction solution.

## 3 Results

### 3.1 XRD characterization

**FIGURE 2:**
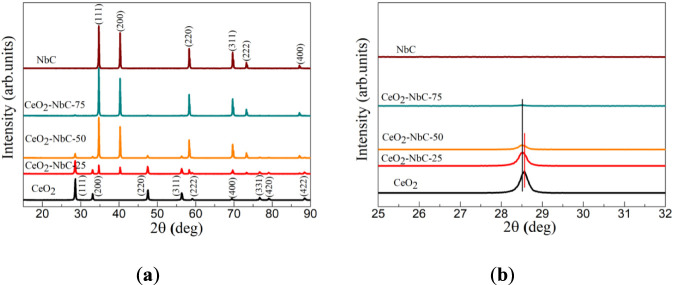
**(a)** XRD patterns and **(b)** enlarged patterns of the CeO_2_, CeO_2_-NbC-25, CeO_2_-NbC-50, CeO_2_-NbC-75 and NbC.

In this experiment, NbC, CeO_2_, and CeO_2_-NbC-25, CeO_2_-NbC-50, CeO_2_-NbC-75 were prepared by the molten salt method, polyacrylamide gel method, and heat treatment technology, respectively. To determine the phase structure and purity of the synthesized target products, detailed analyses were conducted on them using an X-ray diffractometer (XRD). XRD patterns of the CeO_2_, CeO_2_-NbC-25, CeO_2_-NbC-50, CeO_2_-NbC-75 and NbC as shown in Figure 2a. For CO100%, nine obvious diffraction peaks observed, respectively marked as (111), (200), (220), (311), (222), (400), (331), (420) and (422) crystal planes can be assigned to the cubic phase of CeO_2_ with the standard JCPDF no. 89–8,436 and space group of Fm-3 m (225). Characteristic peaks without any impurities were observed. Similarly, six diffraction peaks were observed in the XRD pattern of NbC, corresponding to the (111), (200), (220), (311), (222), and (400) crystal planes of the cubic phase of NbC with the standard JCPDF no.89-3,690 and space group of Fm-3 m (225). With the increase of NbC content, the intensity of the diffraction peak of CeO_2_ in the CeO_2_-NbC-x sample weakens while the intensity of the diffraction peak of NbC increases. Compared with the diffraction peaks of CeO_2_ and NbC, the positions of the diffraction peaks of CeO_2_ and NbC in the CeO_2_-NbC-x sample have slightly shifted towards a lower angle (Figure 2b), suggesting that a special interfacial contact has formed between CeO_2_ and NbC. This conclusion requires further elemental mapping characterization for confirmation.

### 3.2 Surface morphology analysis

The microstructure, particle size distribution, elemental distribution, and elemental composition of the synthesized samples can be observed through scanning electron microscopy (SEM) and transmission electron microscopy (TEM).

**FIGURE 3:**
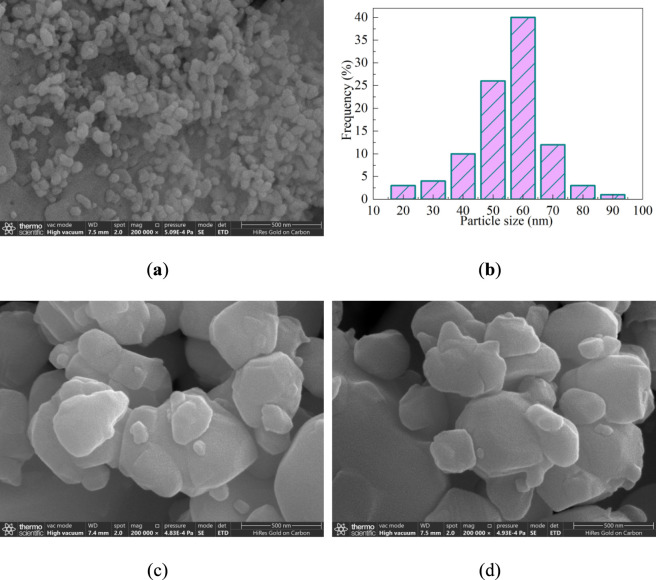
SEM characterization of the CeO_2_, NbC and CeO_2_-NbC-75. **(a)** SEM image and **(b)** Particle size distribution of the CeO_2_; SEM image of **(c)** NbC and **(d)** CeO_2_-NbC-75.

Figure 3a shows the SEM image of the CeO_2_. The CeO_2_ sample is mainly composed of some fine particles that are approximately spherical in shape, and a small amount of adhesion and agglomeration occurred between the particles. The particles are relatively uniform, with an average particle size of approximately 60 nm (Figure 3b). Figure 3c shows the SEM image of the NbC. The NbC sample is mainly composed of some large and fine irregular particles, with the largest particle diameter reaching approximately 500 nm. When 75% of NbC is coupled with CeO_2_, the fine particles slightly increase and adhere to the surface of the large particles as shown in Figure 3d. The CeO_2_ and NbC cannot be distinguished from SEM images, so further analysis through TEM characterization is required. There are mainly three reasons why CeO_2_ and NbC cannot be clearly distinguished: First, the uneven distribution of components or agglomeration phenomena mask the morphology of other components. The second is visual neglect caused by contrast differences. Thirdly, the locality of the imaging area leads to some particles being unable to be observed.

**FIGURE 4:**
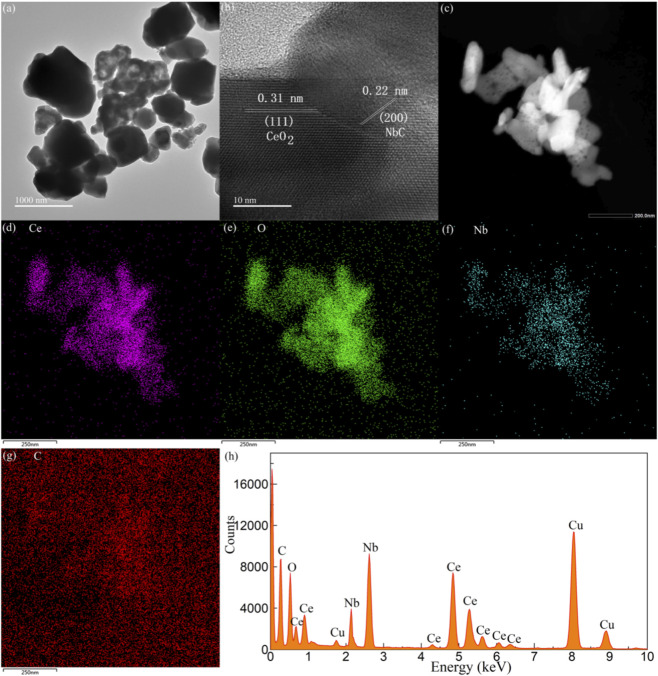
TEM and EDS characterization of CeO_2_-NbC-75. **(a)** TEM image; **(b)** HRTEM image; **(c)** BF-TEM image; Element mapping images of **(d)** Ce, **(e)** O, **(f)** Nb, and **(g)** C; **(h)** EDS spectrum.

Figure 4a shows the TEM image of the CeO_2_-NbC-75. The CeO_2_-NbC-75 sample is composed of large particles and fine particles with obvious agglomeration. The diameter of the largest particle exceeds 500 nm, which is consistent with the results observed by SEM. Due to the obvious agglomeration of fine particles, the spherical outline is not very clear. Figure 4b shows the HRTEM image of the CeO_2_-NbC-75. The lattice fringes between the particles are very clear. The particles with lattice spacings of 0.31 and 0.22 nm are attributed to the (111) crystal plane of CeO_2_ and the (200) crystal plane of the NbC, respectively. The results confirmed that CeO_2_ and NbC were present in the CeO_2_-NbC-75 sample, and the particles formed interfacial contact in a special way. Figures 4c–g shows the BF-TEM and element mapping images of the CeO_2_-NbC-75. All elements are uniformly distributed on the parent body of the NbC, confirming the formation of a heterojunction between CeO_2_ and NbC. To further analyze the purity of the CeO_2_-NbC-75 sample, Figure 4h shows the corresponding EDS spectrum of the CeO_2_-NbC-75. The EDS spectrum contains elements such as C, O, Ce, Nb and Cu. Due to the use of copper mesh in the TEM test process, the sample contains Cu element, so there are no impurity elements in the sample.

### 3.3 Optical properties

Whether semiconductor materials have a high optical absorption coefficient can be used to determine their potential applications in the field of photocatalysis. To study the optical properties of the CeO_2_, NbC, and CeO_2_-NbC-x, they were characterized by an ultraviolet-visible spectrophotometer.

**FIGURE 5:**
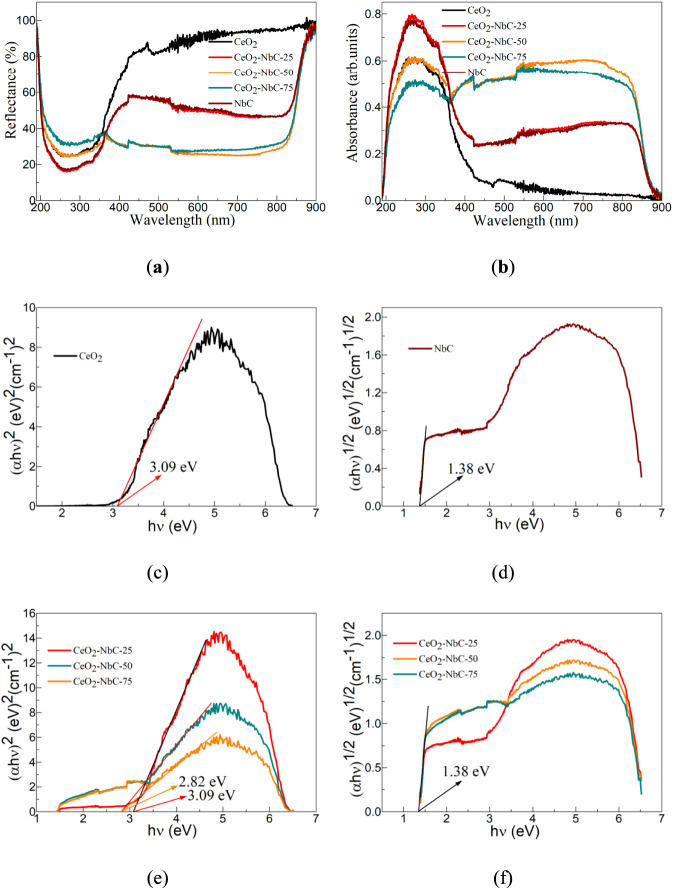
Optical characterization of the CeO_2_, CeO_2_-NbC-25, CeO_2_-NbC-50, CeO_2_-NbC-75 and NbC. **(a)** Diffuse reflection spectrum; **(b)** Absorption spectrum; **(c)** E.g., value of CeO_2_; **(d)** E.g., value of NbC. The curve relationship of **(e)** (αhν)^2^ ∼ hν and **(f)** (αhν)^1/2^ ∼ hν for the CeO_2_-NbC-x catalysts.

Figure 5a shows the diffuse reflection spectrum of the CeO_2_, CeO_2_-NbC-25, CeO_2_-NbC-50, CeO_2_-NbC-75, and NbC. For all samples, the reflectance drops sharply in the range of 190–250 nm, and increases with the increase in wavelength in the range of 250–350 nm. The difference is that after a wavelength of 350 nm, the reflectance of the CeO_2_ sample increases sharply in the range of 350–450 nm and slowly in the range of 460–900 nm. The reflectance of the CeO_2_-NbC-25 and NbC samples increased sharply in the range of 350–420 nm, slightly decreased in the range of 420–800 nm, and increased sharply after exceeding 800 nm. Excluding the influence of the test steps, the reflectance of the CeO_2_-NbC-50 and CeO_2_-NbC-75 samples remained almost constant in the 350–800 nm range and it increased sharply after 800 nm. It is worth noting that after a wavelength of 350 nm, the CeO_2_ sample has the highest reflectance, while the reflectance of the CeO_2_-NbC-25 and NbC samples is similar. The reflectance of the CeO_2_-NbC-50 and CeO_2_-NbC-75 samples is also similar. Within the wavelength range of 500–800 nm, the reflectance of the CeO_2_-NbC-50 sample is the smallest. The reflectance of CeO_2_ and NbC is greater than that of the CeO_2_-NbC-50 and CeO_2_-NbC-75, indicating that the reflectance of the CeO_2_-NbC-x after the coupling of CeO_2_ and NbC is not a simple mechanical superposition of the two.

Based on Kubelka-Munk (K-M) Equation 6 (Singh et al., 2026) and the diffuse reflection spectrum of the CeO_2_, CeO_2_-NbC-25, CeO_2_-NbC-50, CeO_2_-NbC-75, and NbC, the absorption spectra of the CeO_2_, CeO_2_-NbC-25, CeO_2_-NbC-50, CeO_2_-NbC-75, and NbC obtained, as shown in Figure 5b.

$$F\left(R\right)=\frac{\alpha }{S}=\frac{{\left(1-R_{\infty }\right)}^{2}}{2R}$$ (6)

Where, R, α and S is the reflectivity, the absorption coefficient and the scattering coefficient of the CeO_2_, CeO_2_-NbC-25, CeO_2_-NbC-50, CeO_2_-NbC-75, and NbC, respectively. The absorption coefficients and reflectance of all samples show an opposite trend. The CeO_2_ sample has a high ultraviolet optical absorption coefficient, and the NbC sample has a high optical absorption coefficient in both the ultraviolet and visible light wavelength ranges. In the range of 200–350 nm, the absorption coefficient of the CeO_2_-NbC-x decreases with the increase of NbC content. In the range of 350–900 nm, the absorption coefficient of the CeO_2_-NbC-x does not show a significant linear correlation. The absorption band at 200–350 nm can be assigned to the Ce-O centers in CeO_2_ (Zheng et al., 2021). Similar characteristic absorption peaks were also observed in NbC, mainly due to the possible presence of a small amount of C in the sample (Gupta and Pandey, 2019). The absorption observed at 350–900 nm can be associated with the presence of disordered Nb-C centers in NbC (Gupta et al., 2018). Absorption spectrum analysis indicates that the CeO_2_-NbC-x has a high ultraviolet-visible optical absorption coefficient, revealing that it can respond to ultraviolet-visible light.

According to the Tauc relationship (Equation 7) (Escobedo-Morales et al., 2026), the absorption spectra of the CeO_2_, CeO_2_-NbC-25, CeO_2_-NbC-50, CeO_2_-NbC-75, and NbC can be converted into a curve relationship of (αhν)^2^ ∼ hν or (αhν)^1/2^ ∼ hν, as shown in Figures 5c,d.

$${\left(\alpha h\nu \right)}^{n}=A\left(h\nu -E_{g}\right)$$ (7)

Where, h is the Planck constant, ν is the frequency, E.g., is the optical band gap value, and A is a constant. n = 2 and 1/2 is a direct bandgap semiconductor and an indirect bandgap semiconductor, respectively. The CeO_2_ and NbC is a direct bandgap semiconductor (Isik et al., 2023) and an indirect bandgap semiconductor (Sivasankeerthana et al., 2024), respectively. Figure 5c shows the, E.g., value of the CeO_2_. The intersection point value of the slope at the steepest part of the (αhν)^2^ ∼ hν curve and the abscissa is the, E.g., value. According to the calculation, the, E.g., value of the CeO_2_ is approximately 3.09 eV. The, E.g., value of the NbC as shown in Figure 5d. The, E.g., value of the NbC is 1.38 eV, slightly lower than the results reported in the literature (Gurung, 2012). When CeO_2_ and NbC are coupled, the, E.g., value of the host lattice does not change. When the NbC content is low, the, E.g., value of the CeO_2_-NbC-25 catalyst is 3.09 eV, as shown in Figure 5e. When the NbC content exceeds 25%, the, E.g., value of the CeO_2_-NbC-x catalyst is 1.38 eV, as shown in Figure 5f.

### 3.4 Photocatalytic activity

#### 3.4.1 The influence of different catalysts on photocatalytic activity

**FIGURE 6:**
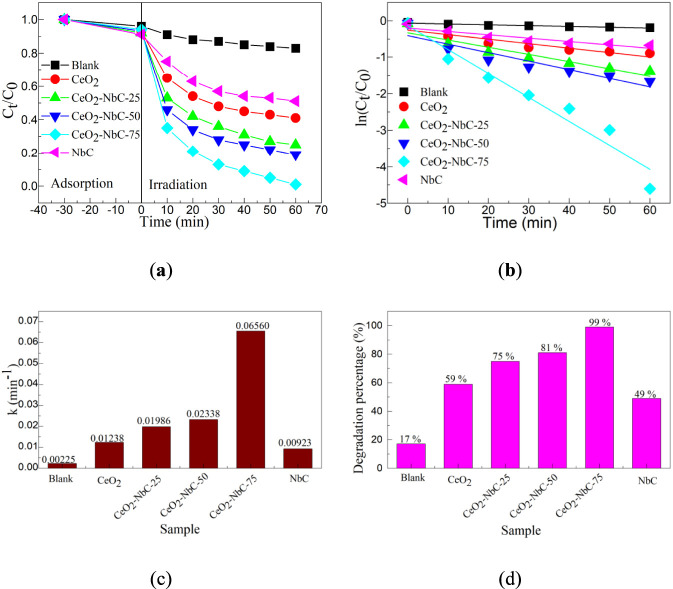
The photocatalytic activity of different catalysts. **(a)** Photocatalytic degradation curve; **(b)** First-order dynamic curve; **(c)** First-order kinetic constants; **(d)** Degradation percentage.

Taking OTC-HCl as the target pollutant, the photocatalytic activity of different catalysts for the degradation of OTC-HCl was explored. Figure 6a shows the photocatalytic degradation curve of different catalysts. Before the photocatalytic experiment, a half-hour adsorption experiment was carried out. The results showed that these catalysts were difficult to adsorb OTC-HCl, and the maximum adsorption percentage did not exceed 10%. In this experiment, a blank experiment was also conducted. Without the participation of a catalyst, it was simulated that sunlight was difficult to degrade OTC-HCl. After 60 min of light exposure, the degradation percentage was only about 17%. Adsorption experiments and blank experiments confirmed that the OTC-HCl is difficult to degrade naturally under environmental conditions. When all the catalysts were exposed to simulated sunlight, the degradation rate increased with the increase in exposure time. The degradation rates of the CeO_2_ and NbC catalysts are relatively low, both not exceeding 60%. The CeO_2_-NbC-x catalyst demonstrated a higher degradation rate than the CeO_2_ and NbC catalysts, and the CeO_2_-NbC-75 catalyst showed the best photocatalytic degradation activity.

To visually reflect the photocatalytic activity of different catalysts, a first-order kinetic curve was introduced to calculate the logarithm of the degradation rate curve (Equation 8) (Hassan et al., 2026).

$$\ln\left(C_{t}/C_{0}\right)=-\text{kt}$$ (8)

Where, C_0_ and C_t_ is the initial concentration and the concentration at time t of the OTC-HCl, respectively. The k is the first-order kinetic constant. Figure 6b shows the first-order dynamic curve of different catalysts. There is a high linear correlation between ln(C_t_/C_0_) and t. The first-order kinetic constants of different catalysts are shown in Figure 6c. The k values of the blank experiment, CeO_2_, CeO_2_-NbC-25, CeO_2_-NbC-50, CeO_2_-NbC-75 and NbC are 0.00225, 0.01238, 0.01986, 0.02338, 0.06560 and 0.00923 min^-1^, respectively. The degradation rate of the CeO_2_-NbC-75 catalyst was 29.16 times that of the blank experiment, 5.30 times that of the CeO_2_ catalyst, and 7.11 times that of the NbC catalyst. The results confirmed that the CeO_2_-NbC-75 catalyst had the best photocatalytic efficiency, and all subsequent photocatalytic experiments were based on this catalyst. The degradation percentage of different catalysts is shown in Figure 6d. The degradation percentages of the blank experiment, CeO_2_, CeO_2_-NbC-25, CeO_2_-NbC-50, CeO_2_-NbC-75, and NbC are 17%, 59%, 75%, 81%, 99%, and 49%, respectively. The results further confirmed that the CeO_2_-NbC-75 catalyst had the best photocatalytic efficiency for the degradation of OTC-HCl.

#### 3.4.2 The influence of different reaction conditions on photocatalytic activity

**FIGURE 7:**
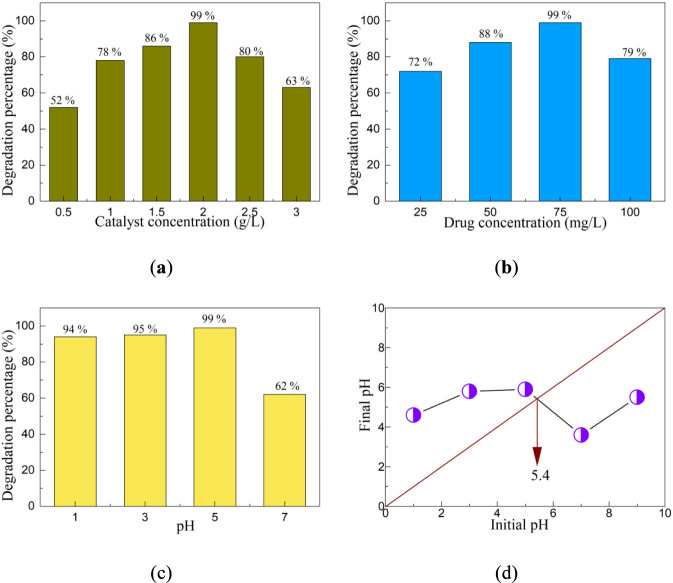
The influence of different reaction conditions on the photocatalytic activity of the CeO_2_-NbC-75 catalyst. **(a)** Catalyst concentration; **(b)** Drug concentration; **(c)** pH value; **(d)** Point of zero charge (PZC).

Different reaction conditions can have a huge impact on the photocatalytic activity of the catalyst. To study the influence of these conditions on the photocatalytic activity of the catalyst, the catalyst concentration, the drug concentration and the pH value of the reaction solution were selected as conditions for detailed exploration. Figure 7a shows the influence of catalyst concentration on the photocatalytic activity of the CeO_2_-NbC-75 catalyst. The catalyst concentration was carried out from 0.5 g/L to 3 g/L, and a photocatalytic experiment was conducted for each increase of 0.5 g/L. During the photocatalytic experiment, except for the change in catalyst concentration, the other experimental processes remain consistent. With the increase of catalyst concentration, the degradation percentage of the CeO_2_-NbC-75 catalyst first increases and then decreases. When the catalyst concentration is low, the degradation percentage of the CeO_2_-NbC-75 catalyst increases with the increase of catalyst concentration. If the catalyst concentration is too low, it will cause excessive pollutants to adhere to the surface of the catalyst, resulting in insufficient active sites of the catalyst and its inability to degrade pollutants (Nejat and Zandi, 2025). When the content of the catalyst continuously increases, the active sites of the catalyst are effectively utilized, resulting in an increase in its degradation percentage. When the catalyst concentration was 2 g/L, the degradation percentage of the CeO_2_-NbC-75 catalyst reached 99%. When the catalyst concentration exceeds 2 g/L, the degradation percentage of the CeO_2_-NbC-75 catalyst drops significantly, mainly due to the excessive amount of catalyst causing the excess photons to not be effectively utilized (Rhaya et al., 2025). Through the research of photocatalytic experiments with different catalyst concentrations, it was found that the optimal catalyst concentration for the degradation of OTC-HCl by the CeO_2_-NbC-75 catalyst is 2 g/L.

The drug concentration is also an important factor affecting the photocatalytic activity of the catalyst. Figure 7b shows the influence of different drug concentrations on the photocatalytic activity of the CeO_2_-NbC-75 catalyst. Similar to the influence law of catalyst concentration on the photocatalytic activity of the catalyst, as the concentration of the drug increases, the degradation percentage of the CeO_2_-NbC-75 catalyst first increases and then decreases. When the concentration of the drug is too low, the effective active sites of the catalyst cannot be effectively utilized, causing its photocatalytic activity to increase with the increase of the drug concentration (Tang et al., 2025). When the concentration of the drug is too high, the excess drug adsorbs on the surface of the catalyst, prolonging the path length for photons to penetrate the reaction solution and reach the surface of the photocatalyst, thereby degrading its photocatalytic activity (Zong et al., 2025). Therefore, the optimal drug concentration for the degradation of OTC-HCl by the CeO_2_-NbC-75 catalyst is 75 mg/L.

Similarly, the pH value of the reaction solution is also one of the important parameters that affect the photocatalytic activity of the catalyst. Figure 7c shows the influence of different pH values on the photocatalytic activity of the CeO_2_-NbC-75 catalyst. In an acidic environment, the CeO_2_-NbC-75 catalyst exhibits high photocatalytic activity. Especially when pH = 5, the degradation percentage of the CeO_2_-NbC-75 catalyst reached 99%. When the pH value is neutral, the degradation percentage of the CeO_2_-NbC-75 catalyst drops sharply to only 62%. The possibility of this situation occurring is related to the point of zero charge (PZC) of the catalyst. The PZC value of the CeO_2_-NbC-75 catalyst as shown in Figure 6d. The PZC value of the CeO_2_-NbC-75 catalyst is 5.4. Čerović et al. (2007) reported that the PZC value of NbC was 3.6, while De Faria and Trasatti. (1994) reported that the PZC value of CeO_2_ was 8.1. The PZC value of the CeO_2_-NbC-75 catalyst is between NbC and CeO_2_, which indicates that the testing method used in this experiment is feasible. According to Equations 9, 10, the surface of the CeO_2_-NbC-75 catalyst becomes positively charged at pH < 5.4 and negatively charged at pH > 5.4.

$$\text{pH }<\text{ PZC}:\left(\text{Ce},\text{Nb}\right)-\text{OH}+H^{+}\;\Leftrightarrow \;\left(\text{Ce},\text{Nb}\right){\text{OH}}_{2}^{+}$$ (9)

$$\text{pH }>\text{PZC}:\left(\text{Ce},\text{Nb}\right)-\text{OH}+{\text{OH}}^{-}\;\Leftrightarrow \;\left(\text{Ce},\text{Nb}\right)O^{-}+H_{2}O$$ (10)

From the perspective of photocatalytic experiments, the CeO_2_-NbC-75 catalyst prefers to degrade OTC-HCl in an acidic environment. When the pH value of the reaction solution is less than 5.4, the surface charge of the CeO_2_-NbC-75 catalyst is positive, and it is prone to react with oxytetracycline hydrochloride. The results confirmed that the optimal pH value for the degradation of OTC-HCl by the CeO_2_-NbC-75 catalyst was 5.

#### 3.4.3 Cyclic and capture experiments

**FIGURE 8:**
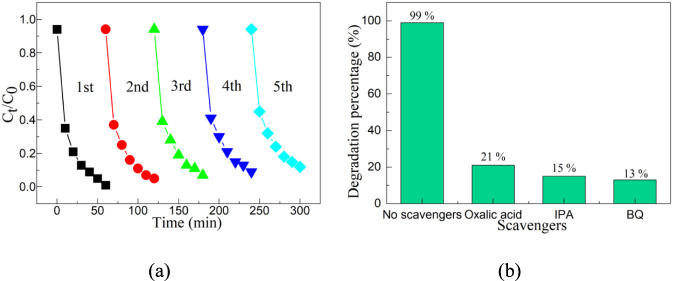
**(a)** Cyclic and **(b)** capture experiments of the CeO_2_-NbC-75 catalyst.

Whether a catalyst can be recycled is an important indicator for evaluating whether it can be industrially applied. Therefore, cyclic stability experiments need to be conducted to confirm that the CeO_2_-NbC-75 catalyst has high cyclic stability. Figure 8a shows the cyclic experiments of the CeO_2_-NbC-75 catalyst. Before the cyclic stability experiment is carried out, the reaction solution from the previous experiment needs to be centrifuged, the catalyst filtered out, and the catalyst dried and sintered at low temperature to remove the surface-adsorbed pollutant molecules. Then, the next photocatalytic experiment can be conducted. After five cycles of experiments, the degradation rate of the CeO_2_-NbC-75 catalyst only slightly decreased. The main reasons for the decline include two: First, the loss of the catalyst during the experiment led to a decrease in catalytic activity. Secondly, after numerous repeated experiments, the partial deactivation of the surface active sites of the catalyst can also lead to a decline in catalytic activity. In conclusion, after multiple cycles of use, it has been confirmed that the CeO_2_-NbC-75 catalyst has high cycling stability.

The CeO_2_-NbC-75 catalyst exhibits high photocatalytic activity, and it is speculated that active species such as holes, hydroxyl radicals, and superoxide radicals play significant roles in the photocatalytic process. An effective way to verify whether active species play an important role in the photocatalytic process is to conduct capture experiments. Oxalic acid, isopropanol (IPA), and benzoquinone (BQ) were used as the scavengers for capturing holes, hydroxyl radicals and superoxide radicals, respectively. Figure 8b shows the capture experiments of the CeO_2_-NbC-75 catalyst. When oxalic acid, IPA, and BQ were added, the photocatalytic activity of the CeO_2_-NbC-75 catalyst was greatly inhibited, and the maximum degradation percentage was only 21%. The results show that the holes, hydroxyl radicals, and superoxide radicals are the main active species for the degradation of OTC-HCl by the CeO_2_-NbC-75 catalyst.

### 3.5 Photocatalytic mechanism

Photocatalytic experiments have confirmed that the CeO_2_-NbC-x catalyst has high photocatalytic activity for the degradation of OTC-HCl. XRD and elemental mapping characterations confirmed the formation of a special interfacial contact between CeO_2_ and NbC. The capture experiment confirmed that the holes, hydroxyl radicals and superoxide radicals are the main active species for the degradation of OTC-HCl by the CeO_2_-NbC-x catalyst. According to reference (Bindumadhavan et al., 2013), heterojunctions constructed by grinding and low-temperature sintering are prone to introducing defects, which contribute to enhancing the photocatalytic activity of the system. In this experiment, the formation of a CeO_2_-NBC-X heterojunction by grinding CeO_2_ and NbC helps to construct a special defect structure, which can promote the photocatalytic degradation of OTC-HCl by the CeO_2_-NBC-X catalyst. Therefore, the photocatalytic mechanism of the CeO_2_-NbC-x catalyst needs to be further plotted in combination with the band arrangement theory. The conduction band potential (E_CB_) and valence band potential (E_VB_) of CeO_2_ and NbC catalysts were calculated through Formulas 11,12, [Disp-formula e12] (Anjitha et al., 2026).

$$E_{\text{CB}}=X-E^{e}-0.5E_{g}$$ (11)

$$E_{\text{VB}}=E_{\text{CB}}+E_{g}$$ (12)

Where, X is the absolute electronegativity of CeO_2_ and NbC catalysts. E^e^ = 4.5 eV. The X of the CeO_2_ and NbC catalysts can be calculated through Formulas 13,14 [Disp-formula e14]

$$X\left(\text{Ce}O_{2}\right)=\sqrt[{3}]{X\left(Ce\right)*X^{2}\left(O\right)}$$ (13)

Where, X(Ce) = 2.19 eV, and X(O) = 7.54 eV.

$$X\left(\text{Nb}C\right)=\sqrt[{2}]{X\left(Nb\right)*X\left(C\right)}$$ (14)

**FIGURE 9:**
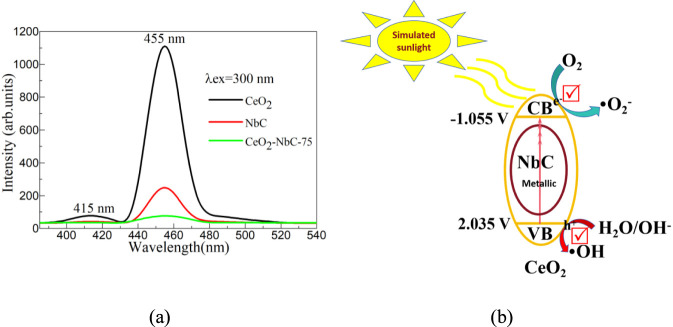
**(a)** Photoluminescence spectra of CeO_2_, NbC and CeO_2_-NbC-75. **(b)** Photocatalytic mechanism of CeO_2_-NbC-75.

Where, X(C) = 6.27 eV, and X(Nb) = 4.0 eV. The X of the CeO_2_ and NbC catalysts is 4.99 and 5.01 V, respectively. According to the calculation, the conduction band potentials of CeO_2_ and NbC are −1.055 and −0.180 V, respectively. The valence band potentials of CeO_2_ and NbC are 2.035 and 1.200 V, respectively. It can be known from the calculation results that after CeO_2_ and NbC couple to form the CeO_2_-NbC-x catalyst, they follow the type I band arrangement. Type I band arrangement helps the recombination of charge carriers, thereby reducing the catalytic activity of CeO_2_-NbC-x catalysts. However, due to the fact that the interface between CeO_2_ and NbC is prone to form an energy barrier (Zhu et al., 2024), it is difficult for electrons to relax from the conduction band of CeO_2_ to that of NbC. Instead, they react with the dissolved oxygen in the reaction solution at the conduction band of CeO_2_ to generate superoxide radicals. The valence band holes of CeO_2_ are prone to transition to the valence band of NbC, but the valence band potential of NbC is only 1.200 V, which is not sufficient for it to react with H_2_O/OH^−^ to form hydroxyl radicals. From this perspective, it is contradictory to the results obtained from the capture experiment. Therefore, it is inappropriate to explain the photocatalytic mechanism of CeO_2_-NbC-x catalysts through type I band arrangement theory. To further explore the charge carrier transfer and separation efficiency of each catalyst, photoluminescence experiments were performed as shown in Figure 9a. Under the light excitation of 300 nm, CeO_2_ can observe two fluorescence emission peaks at 410 and 455 nm. The former is attributed to the transition of electrons between 4f^0^ and 4f^1^, while the latter is attributed to the electronic relaxation of F^0*^ excited State to F^0^ state (Wang et al., 2021). For NbC, only one peak at 455 nm can be observed due to the defect. For CeO_2_-NbC-x catalyst, the fluorescence emission peak is almost quenched. The results show that the CeO_2_-NbC-x catalyst has a high charge carrier transfer and separation efficiency, which is beneficial for the photocatalytic degradation of pollutants. In addition, after CeO_2_ and NbC form the CeO_2_-NBC-X catalyst, a special interface defect is formed at their interface. This interface defect is conducive to the directional transfer and separation of charge carriers. According to the literature (Rahim and Rodriguez, 2013; Zhang et al., 2025), NbC possesses metallic properties, which will enable it to serve as a carrier for charge carrier transport when combined with CeO_2_, promoting charge transfer and separation within CeO_2_ as shown in Figure 9b. The photoluminescence experiment confirmed that this hypothesis was reasonable. When simulated sunlight is shone on the surface of the CeO_2_-NbC-x catalyst, the electrons in the CeO_2_ valence band will be excited and accelerate their transition to the conduction band of the CeO_2_ under the action of NbC, promoting the separation of electrons and holes in CeO_2_. Because the conduction band potential of CeO_2_ is lower than −0.13 V and the valence band potential is greater than 1.89 V, the conduction band electrons are prone to react with oxygen in the solution to form superoxide radicals, and the valence band holes will react with H_2_O/OH^−^ to form hydroxyl radicals. Both the generated superoxide radicals and hydroxyl radicals will undergo degradation reactions with OTC-HCl, generating non-toxic small molecules. In addition, the valence band holes will also directly react with OTC-HCl to form non-toxic small molecules. The specific chemical reactions (Equations 15–20) are as follows:

$${\text{CeO}}_{2}-\text{NbC}-x+h\nu \;\to \;{\text{CeO}}_{2}-\text{NbC}-x\;\left(e_{\text{CB}}^{-}+h_{\text{VB}}^{+}\right)$$ (15)

$$e_{\text{CB}}^{-}+O_{2}\to \cdot O_{2}^{-}$$ (16)

$$h_{\text{VB}}^{+}+{\text{OH}}^{-}\;\to \;\cdot \text{OH}$$ (17)

·OH+drug → Non−toxic small molecules (18)

$$\cdot O_{2}^{-}+\text{drug }\to \text{ Non}-\text{toxic small molecules}$$ (19)

$$h_{\text{VB}}^{+}+\text{drug }\to \text{ Non}-\text{toxic small molecules}$$ (20)

## 4 Discussion

In this study, a heterojunction was designed based on wide bandgap semiconductors by coupling semiconductor materials with metallic properties to enhance the visible light response capability of wide bandgap semiconductors. NbC has an optical bandgap value of 1.38 eV, but it also possesses metallic properties, which enable it to form a heterojunction with a type I band arrangement structure during the coupling process with CeO_2_, while also acting as a metal to accelerate the transport of electrons and holes in CeO_2_. Such a special structure facilitates the transfer and separation of electron-hole pairs, thereby enhancing the photocatalytic activity of wide bandgap semiconductor materials. This idea has broadened the boundaries for the application of new wide bandgap semiconductor materials in the field of photocatalysis. This experiment only used OTC-HCl as the target pollutant for degradation experiments. Subsequent experiments can also explore the application of similar catalysts in the degradation of dyes, other drugs and refractory pollutants.

During the photocatalytic experiment, each researcher adopted different experimental conditions to carry out the photocatalytic experiment. It is extremely difficult to truly compare the photocatalytic degradation efficiency of different catalysts and under different reaction conditions. This brings considerable difficulty to judging the photocatalytic activity and the speed of degradation of the synthesized photocatalyst. This difficult problem can be effectively solved by introducing the concept of specific activity (SA) (Equation 21).

$$\text{SA}=\left(C_{\text{Drug}}\times D\right)\;/\;\left(C_{\text{Catalyst}}\times t\right)$$ (21)

Where, C_Drug_ represents the concentration of the drug, D represents the percentage of degradation, C_Catalyst_ represents the content of the catalyst, and t represents the reaction time. Table 1 given the comparisons of OTC-HCl degraded by different photocatalysts (Dai et al., 2019; Feng et al., 2025; Hong et al., 2019; Mahmoudi et al., 2024; Majumdar et al., 2021; Ouyang et al., 2021; Vaizoğullar, 2019; Xu et al., 2020). By calculating the SA values of different catalysts, it was found that the photocatalyst prepared in this experiment has a relatively large SA value. Although the BP/Bi_2_MoO_6_ photocatalyst prepared by Feng et al. (2025) had the highest SA value, H_2_O_2_ was added during the photocatalytic reaction process, which does not represent the true degradation efficiency of the BP/Bi_2_MoO_6_ photocatalyst for OTC-HCl. From this perspective, the CeO_2_-NC-75 photocatalyst obtained in this experiment demonstrated the best degradation efficiency in the degradation of OTC-HCl.

**TABLE 1:** Comparisons of OTC-HCl degraded by different photocatalysts.

Photocatalysts	Light source	*Catalyst content* (g/L)	*Drug concentration* (mmol/L)	t (h)	D (%)	Specific activity (mmol g/h)	Ref.
Ag/AgCl/BiVO_4_	Xenon lamp (1000 W)	1	0.20 (20 mg/L)	2	97.6	0.0976	Dai et al. (2019)
Ag/BiVO_4_/GO	Xenon lamp (500 W)	0.4	0.20	1.17	82.65	0.3532	Ouyang et al. (2021)
BP/Bi_2_MoO_6_ + H_2_O_2_	White LED source (40 W)	0.5	0.20	0.67	92.9	0.5546	Feng et al. (2025)
g-C_3_N_4_/Fe_3_O_4_	UV-A lamp (15 W)	0.7	0.05	1	99.8	0.0713	Mahmoudi et al. (2024)
LaFeO_3_/g-C_3_N_4_	White LED (40 W)	0.5	0.40	2	90.0	0.3600	Xu et al. (2020)
g-C_3_N_4_/Bi_4_NbO_8_Cl	Visible LED light (18 W)	1	0.2	1	87.0	0.1740	Majumdar et al. (2021)
ZnO/ZrO_2_	UV	24	0.1	2	69.0	0.0014	Vaizoğullar (2019)
Br (15%)/g-C_3_N_4_	White LED (38.5 W)	1	0.1	2	75.0	0.0375	Hong et al. (2019)
CeO_2_-NbC-75	Xenon lamp (300 W)	2	0.75	1	99	0.3713	This work

Although a reasonable photocatalytic mechanism has been proposed, a more instructive mechanism for experiments can be put forward by combining first-principles calculations. The development of new catalysts through the combination of experiments and theories broadens the path for new applications in the field of photocatalysis. Compared with other similar photocatalysts (Chin et al., 2023; Singh et al., 2021; Wang et al., 2023), CeO_2_-NbC-x catalyst has a more rapid degradation efficiency and can better stimulate its potential for industrial applications. Moreover, this catalyst does not have side effects such as hydrolysis, making its application in the field of photocatalysis effortless. This is a very promising technical means to achieve efficient degradation of pollutants by photocatalysts.

With the continuous development of artificial intelligence technology, introducing relevant intelligent algorithms to train and predict the experimental and theoretical data of wide bandgap semiconductor-based catalysts, and obtaining the optimal new catalysts, has become an urgent problem to be solved at present. By constructing special algorithm models to screen new catalysts, experimental costs can be significantly reduced, the number of trial-and-error attempts can be decreased, and thus labor costs can be saved and development time shortened. This is also a current research hotspot in the field of catalysis, which can significantly shorten the development time of catalysts for efficiently degrading pollutants and may also promote new developments in artificial intelligence technology.

## 5 Conclusion

The CeO_2_@NbC catalyst for the efficient degradation of OTC-HCl was synthesized by the molten salt method, polyacrylamide gel method and heat treatment technology. XRD characterization determined that the CeO_2_-NbC-x catalyst contained only CeO_2_ and NbC phases, without any other impurities. SEM and TEM characterizations confirmed the formation of a special heterojunction between the CeO_2_ and NbC interfaces. The optical property characterization confirmed that the, E.g., values of the CeO_2_ and NbC were 3.09 and 1.38 eV, respectively, and the coupling of the two demonstrated a high ultraviolet-visible optical absorption coefficient. When the optimal NbC content, catalyst concentration, pollutant concentration and pH value are 75%, 2 g/L, 75 mg/L and 5, respectively, the CeO_2_-NbC-x catalyst exhibits a high degradation percentage (99%) for the degradation of OTC-HCl. Based on the capture experiment and photocatalytic mechanism analysis, holes, hydroxyl radicals and superoxide radicals are the main active species in the degradation of OTC-HCl by CeO_2_-NbC-x catalyst. This heterojunction enables the rapid degradation of OTC-HCl, which is conducive to its extended application in the design of other similar catalysts.

## Data Availability

The original contributions presented in the study are included in the article/supplementary material, further inquiries can be directed to the corresponding author.

## References

[B1] Ali M. M. Zhang L. Xu Y. Gaballah M. S. Gaballah E. S. Samer M. (2025). Nexus between anaerobic digestion of animal waste and antibiotic-related pollutants: a critical review. Appl. Energ. 382, 125284. 10.1016/j.apenergy.2025.125284

[B2] Anjitha A. Leen S. Zielińska-Jurek A. Sridharan K. (2026). Phase-engineered bismuth-rich oxybromides (Bi_x_O_y_Br_z_) for visible-light photocatalytic degradation of emerging pollutants and harmful algal blooms. Nanoscale. 10.1039/D5NR05038C 41562345

[B3] Ansari A. A. Lv R. Gai S. Yang P. (2025). Chemistry of CeO_2_-derived nanocomposites photocatalysts for environment monitoring and energy conversion. Chin. J. Catal. 71, 70–113. 10.1016/S1872-2067(24)60266-4

[B4] Baaloudj O. Assadi I. Nasrallah N. El Jery A. Khezami L. Assadi A. A. (2021). Simultaneous removal of antibiotics and inactivation of antibiotic-resistant bacteria by photocatalysis: a review. J. Water Process Eng. 42, 102089. 10.1016/j.jwpe.2021.102089

[B5] Bindumadhavan K. Srivastava S. K. Mahanty S. (2013). MoS_2_–MWCNT hybrids as a superior anode in lithium-ion batteries. Chem. Commun. 49 (18), 1823–1825. 10.1039/C3CC38598A 23358567

[B6] Čerović L. S. Milonjić S. K. Todorović M. B. Trtanj M. I. Pogozhev Y. S. Blagoveschenskii Y. (2007). Point of zero charge of different carbides. Colloid. Surf. A 297 (1-3), 1–6. 10.1016/j.colsurfa.2006.10.012

[B7] Chen Y. Zhang H. Zhang J. Ma J. Wang L. Ye H. (2013). Facile synthesis, characterization and photocatalytic activity of niobium carbide. Adv. Powder Technol. 24 (1), 207–211. 10.1016/j.apt.2012.06.002

[B8] Cheng M. Li H. Wu Z. Yu Z. Tao X. Huang L. (2025). Synergistic effects of CQDs and oxygen vacancies on CeO_2_ photocatalyst for efficient photocatalytic nitrogen fixation. Sep. Purif. Technol. 354, 129299. 10.1016/j.seppur.2024.129299

[B9] Chin J. Y. Ahmad A. L. Low S. C. (2023). Graphitic carbon nitride photocatalyst for the degradation of oxytetracycline hydrochloride in water. Mater. Chem. Phys. 301, 127626. 10.1016/j.matchemphys.2023.127626

[B10] Choudhary V. Vellingiri K. Thayyil M. I. Philip L. (2021). Removal of antibiotics from aqueous solutions by electrocatalytic degradation. Environ. Sci. Nano 8 (5), 1133–1176. 10.1039/D0EN01276A

[B11] Dai Y. Liu Y. Kong J. Yuan J. Sun C. Xian Q. (2019). High photocatalytic degradation efficiency of oxytetracycline hydrochloride over Ag/AgCl/BiVO_4_ plasmonic photocatalyst. Solid State Sci. 96, 105946. 10.1016/j.solidstatesciences.2019.105946

[B12] De Faria L. A. Trasatti S. (1994). The point of zero charge of CeO_2_ . J. Colloid Interf. Sci. 167 (2), 352–357. 10.1006/jcis.1994.1370

[B13] Deng S. Wang H. Dong Y. Cheng W. Lin X. Wu L. (2025). Morphology effect on the photocatalytic performance of CeO_2_ and insights into the degradation mechanism of tetracycline. J. Environ. Chem. Eng. 13 (1), 115216. 10.1016/j.jece.2024.115216

[B14] Du J. Sun X. Wang S. Yi Z. Liu G. Yang H. (2026). Boosting photocatalytic synthesis of H_2_O_2_ *via* p-type Schottky junction-mediating free electron and photoelectron transfer behavior. Appl. Surf. Sci. 719, 165024. 10.1016/j.apsusc.2025.165024

[B15] Dutta S. Srivastava S. K. Gupta A. K. (2021). Polypyrrole–polyaniline copolymer coated green rice husk ash as an effective adsorbent for the removal of hexavalent chromium from contaminated water. Mater. Adv. 2 (7), 2431–2443. 10.1039/D0MA00862A

[B16] Escobedo-Morales A. Pedraza-Chan M. S. Ruiz-López I. I. Anota E. C. Salazar-Villanueva M. Cortés-Arriagada D. (2026). Accurate determination of the band gap energy of non-translucent semiconductor materials through the Tauc method: theoretical framework, limitations, technical hints, and automated algorithms. Next Mater 10, 101412. 10.1016/j.nxmate.2025.101412

[B17] Feng J. Li X. Ran X. Wang L. Xiao B. Li R. (2025). Enhanced photo-fenton removal of oxytetracycline hydrochloride *via* BP/Bi_2_MoO_6_ Z-scheme heterojunction photocatalyst. Int. J. Mol. Sci. 26 (16), 7751. 10.3390/ijms26167751 40869070 PMC12386328

[B18] Gupta A. Pandey O. P. (2018). Visible irradiation induced photodegradation by NbC/C nanocomposite derived from smoked cigarette litter (filters). Sol. Energy 163, 167–176. 10.1016/j.solener.2017.12.033

[B19] Gupta A. Pandey O. P. (2019). NbC/C heterojunction for efficient photodegradation of methylene blue under visible irradiation. Sol. Energy 183, 398–409. 10.1016/j.solener.2019.03.040

[B20] Gupta A. Mittal M. Singh M. K. Suib S. L. Pandey O. P. (2018). Low temperature synthesis of NbC/C nano-composites as visible light photoactive catalyst. Sci. Rep. 8 (1), 13597. 10.1038/s41598-018-31989-z 30206350 PMC6133931

[B21] Gupta A. Brar L. K. Pandey O. P. (2019). Influence of laboratory and waste grade cellulose acetate on photo and electrocatalytic properties of NbC_x_O_y_/C and NbC/C nanocomposites. Sol. Energy 189, 120–130. 10.1016/j.solener.2019.07.052

[B22] Gurung S. (2012). FP-LAPW calculations of electronic band structure of NbC and NbN. Sci. Vis. 12 (2), 79–82. Available online at: https://sciencevision.org/storage/journal-articles/February2019/1Dy093l8gR4R3zCbwrPW.pdf (Accessed February 27, 2026).

[B23] Hassan R. Abdel-Rahim R. D. Gouda G. A. Nagiub A. M. (2026). Hydrothermal synthesis and optimization of hierarchical copper molybdate nanostructures for photocatalytic degradation of crystal violet and antimicrobial applications. Sci. Rep. 16, 1963. 10.1038/s41598-025-32124-5 41530244 PMC12804913

[B24] Hong J. Hwang D. K. Selvaraj R. Kim Y. (2019). Facile synthesis of Br-doped g-C_3_N_4_ nanosheets *via* one-step exfoliation using ammonium bromide for photodegradation of oxytetracycline antibiotics. J. Indust. Eng. Chem. 79, 473–481. 10.1016/j.jiec.2019.07.024

[B25] Hu F. Zhao S. Yin X. (2018). Size-controllable synthesis of CeO_2_ nanoparticles *via* microwave assisted acrylamide gel method and their fluorescent properties. J. Mater. Sci. Mater. Electron. 29 (19), 16747–16757. 10.1007/s10854-018-9768-7

[B26] Isik M. Delice S. Gasanly N. M. (2023). Temperature dependence of band gap of CeO_2_ nanoparticle photocatalysts. Phys. E 150, 115712. 10.1016/j.physe.2023.115712

[B27] Li X. Zhang X. Song Z. Wang C. Bai J. Shen J. (2025). CeO_2_ nanoparticles dotted on NiAl-LDHs as Z-scheme heterojunction: synergistic enhancement of adsorption and photocatalytic properties. Adv. Comp. Hybrid. Mater. 8 (5), 350. 10.1007/s42114-025-01424-9

[B28] Limbu S. M. Chen L. Q. Zhang M. L. Du Z. Y. (2021). A global analysis on the systemic effects of antibiotics in cultured fish and their potential human health risk: a review. Rev. Aquacult. 13 (2), 1015–1059. 10.1111/raq.12511

[B29] Lulijwa R. Rupia E. J. Alfaro A. C. (2020). Antibiotic use in aquaculture, policies and regulation, health and environmental risks: a review of the top 15 major producers. Rev. Aquacult. 12 (2), 640–663. 10.1111/raq.12344

[B30] Luo Q. Wang Q. Yang X. Yang J. Jiang C. Cui S. (2026). Construction of CeO_2_/MIL-100 (Fe) heterojunction with multi-oxidation-reduction cycle system for photo-Fenton degradation of tetracycline. Mater. Res. Bull. 198, 114026. 10.1016/j.materresbull.2026.114026

[B31] Ma Q. Ren J. Sun X. Chen X. Liu G. Wang S. (2025). Strong evidence for interface-field-induced photocarrier separation in new AgFeO_2_-BiVO_4_ heterostructures and their efficient photo-Fenton degradation of ciprofoxacin. Appl. Surf. Sci. 679, 161275. 10.1016/j.apsusc.2024.161275

[B32] Mahmoudi K. Farzadkia M. Kalantary R. R. Sobhi H. R. Yeganeh M. Esrafili A. (2024). Efficient removal of oxytetracycline antibiotic from aqueous media using UV/g-C_3_N_4_/Fe_3_O_4_ photocatalytic process. Heliyon 10 (9), e30604. 10.1016/j.heliyon.2024.e30604 38765134 PMC11098847

[B33] Majumdar A. Ghosh U. Pal A. (2021). Novel 2D/2D g-C_3_N_4_/Bi_4_NbO_8_Cl nano-composite for enhanced photocatalytic degradation of oxytetracycline under visible LED light irradiation. J. Colloid Interf. Sci. 584, 320–331. 10.1016/j.jcis.2020.09.101 33070072

[B34] Mangla D. Sharma A. Ikram S. (2022). Critical review on adsorptive removal of antibiotics: present situation, challenges and future perspective. J. Hazard. Mater. 425, 127946. 10.1016/j.jhazmat.2021.127946 34891019

[B35] Mousavi S. M. Mohtaram M. S. Rasouli K. Mohtaram S. Rajabi H. Sabbaghi S. (2025). Efficient visible-light-driven photocatalytic degradation of antibiotics in water by MXene-derived TiO_2_-supported SiO_2_/Ti_3_C_2_ composites: optimisation, mechanism and toxicity evaluation. Environ. Pollut. 367, 125624. 10.1016/j.envpol.2024.125624 39746638

[B36] Nadjia L. Abdelkader E. (2025). Design, synthesis and characterization of ceria: assessment of crystallite size and intrinsic strain using XRD profile analysis and its photocatalytic applications. J. Iran. Chem. Soc. 22 (2), 297–324. 10.1007/s13738-024-03149-w

[B37] Nejat R. Zandi S. (2025). Visible-light responsive La_0.7_Sr_0.3_MnO_3_@ TiO_2_/g-C_3_N_4_ nanocomposite for photocatalytic antibiotic degradation and bioactivity applications. J. Alloy. Compd. 1036, 181866. 10.1016/j.jallcom.2025.181866

[B38] Ouyang K. Yang C. Xu B. Wang H. Xie S. (2021). Synthesis of novel ternary Ag/BiVO_4_/GO photocatalyst for degradation of oxytetracycline hydrochloride under visible light. Colloid. Surf. A 625, 126978. 10.1016/j.colsurfa.2021.126978

[B39] Rahim G. P. A. Rodriguez J. A. (2013). Structural and electronic properties of ScC and NbC: a first principles study. Solid State Phenom. 194, 276–279. 10.4028/www.scientific.net/SSP.194.276

[B40] Rhaya M. Abou Oualid H. Malekshah R. E. Ennasraoui B. Ighnih H. Ouachtak H. (2025). Efficient photocatalytic removal of ciprofloxacin antibiotic by dual Z-scheme Ag_3_PO_4_/g-C_3_N_4_/Bi_2_WO_6_ under solar irradiation. J. Water Proc. Eng. 77, 108394. 10.1016/j.jwpe.2025.108394

[B41] Singh R. V. Bhat A. A. Watanabe S. Rao T. G. Lee J. K. Singh V. (2026). Sol–gel synthesized Mn^4+^-doped Ba_2_CaWO_6_ phosphors: structural and EPR studies highlighting luminescence potential. Ceram. Int. 10.1016/j.ceramint.2026.01.210

[B42] Shi W. Sun X. Xu M. Wang S. Liu G. Yang H. (2024). Design of new CoFe_2_O_4_/MXene/NaTaO_3_ double heterostructures for efficient photodegradation of antibiotic. J. Water Process Eng. 67, 106229. 10.1016/j.jwpe.2024.106229

[B43] Singh J. Juneja S. Soni R. K. Bhattacharya J. (2021). Sunlight mediated enhanced photocatalytic activity of TiO_2_ nanoparticles functionalized CuO-Cu_2_O nanorods for removal of methylene blue and oxytetracycline hydrochloride. J. Colloid Interf. Sci. 590, 60–71. 10.1016/j.jcis.2021.01.022 33524721

[B44] Sivasankeerthana M. V. Balavijayalakshmi J. Sakunthala A. (2024). Strain-dependent structural and electronic properties of NbC using first-principles calculations. Int. Conf. Recent Adv. Mater. Sci. Technol. 414, 11–21. 10.1007/978-3-031-69970-2_2

[B45] Soorya K. K. Singh A. Srivastava S. K. Bhattacharya A. Bhatnagar A. Gupta A. K. (2025). Fabrication of 2D/2D Bi_2_MoO_6_/S_x_@gC_3_N_(4−y)_ type-II heterojunction photocatalyst for enhanced visible-light-mediated degradation of tetracycline in wastewater. Dalton Trans. 54 (6), 2403–2420. 10.1039/D4DT02334J 39714918

[B46] Srivastava S. K. (2026). Recent advances on hollow and core-shell intrinsically conducting polymers for their applications in electromagnetic interference shielding/microwave absorption, removal of metal ions/dyes and supercapacitors. RSC Appl. Polym. 4, 120–199. 10.1039/d5lp00230c

[B47] Sun X. Zhang J. Luo M. Ma J. Xian T. Liu G. (2024). Elevating photocatalytic H_2_ evolution over ZnIn_2_S_4_@ Au@ Cd_0.7_Zn_0.3_S multilayer nanotubes *via* Au-mediating H–S antibonding-orbital occupancy. Chem. Eng. J. 499, 156455. 10.1016/j.cej.2024.156455

[B48] Tang H. Hengchao E. Yao C. Wang X. Zhou J. Song W. (2025). Boosted antibiotic elimination over 2D/2D mesoporous CeO_2_/BiOCl S-scheme photocatalyst. Sep. Purif. Technol. 354, 128977. 10.1016/j.seppur.2024.128977

[B49] Vaizoğullar A. I. (2019). ZnO/ZrO_2_ composites: synthesis characterization and photocatalytic performance in the degradation of oxytetracycline antibiotic. Mater. Technol. 34 (8), 433–443. 10.1080/10667857.2019.1574287

[B50] Wang S. Tang S. Gao H. Fang L. Hu Q. Sun G. (2021). Modified polyacrylamide gel synthesis of CeO_2_ nanoparticles by using cerium sulfate as metal source and its optical and photoluminescence properties. J. Mater. Sci. Mater. Electron. 32 (8), 10820–10834. 10.1007/s10854-021-05740-w

[B51] Wang S. Liu H. Zhang Y. Yu X. Han Y. Gao H. (2023). Construction of g-C_3_N_4_/Au/MgAl_2_O_4_ photocatalysts with different coupling methods to improve the photodegradation behavior and performance prediction. J. Environ. Chem. Eng. 11 (6), 111453. 10.1016/j.jece.2023.111453

[B52] Xu K. Yang X. Ruan L. Qi S. Liu J. Liu K. (2020). Superior adsorption and photocatalytic degradation capability of mesoporous LaFeO_3_/g-C_3_N_4_ for removal of oxytetracycline. Catalysts 10 (3), 301. 10.3390/catal10030301

[B53] Yang L. X. Zhang H. L. Wang Y. Liu H. J. Zeng C. L. (2019). A novel and simple method for large-scale synthesis of nanosized NbC powder by disproportionation reaction in molten salt. Ceram. Int. 45 (3), 3791–3796. 10.1016/j.ceramint.2018.11.047

[B54] Yang J. Tian S. Song Z. Hao Y. Lu M. (2025). Recent advances in sorption-based photocatalytic materials for the degradation of antibiotics. Coord. Chem. Rev. 523, 216257. 10.1016/j.ccr.2024.216257

[B55] Zhang Y. Zhao G. Chen Z. Lian H. Gan L. Pan M. (2022). Hierarchically nanostructured Ag/ZnO/nBC for VOC photocatalytic degradation: dynamic adsorption and enhanced charge transfer. J. Environ. Chem. Eng. 10 (6), 108690. 10.1016/j.jece.2022.108690

[B56] Zhang J. Sun X. Zhu W. Liu G. Xian T. Yang H. (2024). Design of CdZnS/BiOCl heterostructure as a highly-efficient piezo-photocatalyst for removal of antibiotic. J. Environ. Chem. Eng. 12 (6), 114405. 10.1016/j.jece.2024.114405

[B57] Zhang T. Sun W. Chen J. Chen J. Li Y. (2025). Interface oxygen vacancy-enhanced core-shell composite material CeO_2_@ SrTiO_3_ utilizes Z-scheme heterojunction to achieve efficient degradation of tetracycline antibiotics. J. Environ. Chem. Eng. 13 (6), 120289. 10.1016/j.jece.2025.120289

[B58] Zheng X. Mofarah S. S. Cazorla C. Daiyan R. Esmailpour A. A. Scott J. (2021). Decoupling the impacts of engineering defects and band gap alignment mechanism on the catalytic performance of holey 2D CeO_2-x_‐based heterojunctions. Adv. Funct. Mater. 31 (38), 2103171. 10.1002/adfm.202103171

[B59] Zhu J. Cheng X. Cui Y. Chen F. (2024). Photocatalytic activity and mechanism of YMnO_3_/NiO photocatalyst for the degradation of oil and gas field wastewater. Front. Chem. 12, 1408961. 10.3389/fchem.2024.1408961 38752200 PMC11094212

[B60] Zong Q. Wei J. Ji H. Chen C. Song Z. Yang T. (2025). Oxygen-vacancy-rich CeO_2_ nanoparticles coating on NH_2_-MIL-68 (In) for efficient photocatalytic antibiotic degradation: performance, degradation pathway, and mechanism insight. J. Alloy. Compd. 1044, 184391. 10.1016/j.jallcom.2025.184391

[B61] Zulfiqar N. Nadeem R. Musaimi O. A. (2024). Photocatalytic degradation of antibiotics *via* exploitation of a magnetic nanocomposite: a green nanotechnology approach toward drug-contaminated wastewater reclamation. ACS Omega 9 (7), 7986–8004. 10.1021/acsomega.3c08116 38405456 PMC10882661

